# Optimization of Melanin Production by *Streptomyces antibioticus* NRRL B-1701 Using *Arthrospira (Spirulina) platensis* Residues Hydrolysates as Low-Cost L-tyrosine Supplement

**DOI:** 10.3390/biotech12010024

**Published:** 2023-03-20

**Authors:** Oranit Kraseasintra, Sritip Sensupa, Kanjana Mahanil, Sada Yoosathaporn, Jeeraporn Pekkoh, Sirasit Srinuanpan, Wasu Pathom-aree, Chayakorn Pumas

**Affiliations:** 1 Department of Biology, Faculty of Science, Chiang Mai University, Chiang Mai 50200, Thailand; 2 Doctor of Philosophy Program in Applied Microbiology (International Program) in Faculty of Science, Chiang Mai University, Chiang Mai 50200, Thailand; 3 Environmental Science Research Centre, Faculty of Science, Chiang Mai University, Chiang Mai 50200, Thailand; 4 Research Center of Microbial Diversity and Sustainable Utilization, Faculty of Science, Chiang Mai University, Chiang Mai 50200, Thailand; 5 Research Center in Bioresources for Agriculture, Industry and Medicine, Department of Biology, Faculty of Science, Chiang Mai University, Chiang Mai 50200, Thailand

**Keywords:** leftover organic material, cyanobacteria, PC extraction, CCD, hydrolysate, novelty supplement, *Streptomyces* metabolites

## Abstract

Melanin is a functional pigment that is used in various products. It can be produced by *Streptomyces antibioticus* NRRL B-1701 when supplemented with L-tyrosine. *Arthrospira (Spirulina) platensis* is a cyanobacterium with high protein content, including the protein phycocyanin (PC). During PC’s extraction, biomass residues are generated, and these residues still contain various amino acids, especially L-tyrosine, which can be used as a low-cost supplement for melanin production. Thus, this study employed a hydrolysate of *A. platensis* biomass residue for L-tyrosine substitution. The effects of two drying methods, namely, lyophilization and dying via a hot air oven, on the proximate composition and content of L-tyrosine in the biomass residue were evaluated. The highest L-tyrosine (0.268 g L-tyrosine/100 g dried biomass) concentration was obtained from a hot-air-oven-dried biomass residue hydrolysate (HAO-DBRH). The HAO-DBRH was then used as a low-cost L-tyrosine supplement for maximizing melanin production, which was optimized by the response surface methodology (RSM) through central composite design (CCD). Using the RSM–CCD, the maximum level of melanin production achieved was 0.24 g/L, which is approximately four times higher than it was before optimization. This result suggests that *A. platensis* residue hydrolysate could be an economically feasible and low-cost alternative source of L-tyrosine for the production of melanin.

## 1. Introduction

Melanin is a class of heterogeneous functional polymeric compounds produced by species from various kingdoms, including bacteria, fungi, animals, and plants. It is a biomacromolecule with a high molecular weight (318.3 g/mol) that is produced by the oxidative polymerization of phenolic or indolic molecules with a negatively charged hydrophobic polymer that is insoluble in both water and chemical solvents [1,2]. Melanin is an effective metal ion chelator, free radical scavenger, and absorber of UV–visible and visible light radiation. Melanin compounds have also been widely exploited for the development of innovative adhesive biomaterials and green bioelectronics due to their hybrid ionic–electronic conductance and redox reversibility features [3]. Additionally, melanin has been employed to create novel, biodegradable, and biocompatible medical devices; nanoparticles; antibacterial drugs; antiviral and anticancer medicines; and radio-protective and antioxidant compounds. This is because they have excellent biocompatibility and biostability, are not cytotoxic, and do not provoke antigenic reactions. Due to melanin’s many beneficial properties, it has a wide range of applications in the agricultural, cosmetics, and medical industries [4].

Currently, melanin has been mostly obtained from marine sources, for instance, the ink sac of the cuttlefish *Sepia officinalis.* However, these approaches are often expensive, unsustainable, and highly dependent on the supply of such substances [5]. On the other hand, it is generally agreed that the biotechnological methods of producing melanin from microorganisms are more scalable and environmentally friendly [6]. Numerous bacteria, including *Streptomyces glaucescens*, *Nocardiopsis alba*, and *Pseudomonas stutzeri*, and fungi, including *Armillaria ostoyae*, *Aspergillus fumigatus*, and *Daldinia concentrica*, have been reported to produce melanin. The duration of microbial melanin production in culture can range from a few days to several weeks and is influenced by factors such as the type of microorganism, growth conditions, and the production method. The yield of melanin can vary greatly, ranging from milligrams per liter to grams per liter, depending on the strain and growth conditions [6,7].

*Streptomyces* strains produce melanins as primary pigments during the secondary metabolism phase [8], but so far, melanin production by these bacteria has only been documented in a few research studies due to the lengthy processing times required, which can take anywhere between 120 and 168 h. The reported melanin yields range from 0.12 to 5.30 g/L according to the different culture conditions [9,10,11,12,13,14]. High productivity is needed for the feasible, large-scale application of biomaterials, including melanin. However, the amount of the substrate (L-tyrosine or L-DOPA) in the growth media used for tyrosinase determines the yield of melanin production [15] from different bacteria, such as the soil bacterial isolates *Bacillus safensis* [16] and *Brevundimonas* sp. SGJ [17], and some fungi, such as *Auricularia auricula* [18] and *Aspergillus nidulans* [19]. Therefore, increasing melanin production efficiency can be accomplished via the natural selection of active enzymes or growth optimization.

Cyanobacteria, or blue-green algae, have recently risen to prominence in microbial research due to their ability to grow in non-potable water without competing with food crops for fertile soil and their contribution to reducing greenhouse gases in the environment through photosynthesis. Additionally, cyanobacterial biomass can be transformed into various high-value products, including biofuels, cosmetics, foods, nutraceuticals, and pharmaceutical compounds [20]. *A. platensis* is one of the most important cyanobacteria genera and is well known for being a high source of protein (up to 50–70% of its cell mass) and bioactive ingredients such as vitamins, β-carotene, micro- and macro-elements, and polyunsaturated fatty acids [21,22]. Phycocyanin (PC) is a mainly water-soluble pigment protein (8–13% of dry biomass) that is found in cyanobacteria, particularly in *A. platensis* [23]. PC sparked attention because of its exceptional capability to exhibit antioxidant, anti-inflammatory, and anticarcinogenic properties. Furthermore, research has shown that it possesses anti-mutative, anti-microbial, antitumor, and wound-healing properties, and it has found applications as food coloring, in cosmetics, and as a dietary supplement [24,25].

The residue biomass left over after PC extraction from *A. platensis* is still rich in valuable compounds such as proteins, carbohydrates, and lipids. This residue can be used for various purposes, including as livestock feed, fertilizer, or feedstock for biofuel production. Furthermore, some studies have investigated the production of crude bio-oil from biomass residues, and it has been discovered that nitrogen reduction via the elimination of pigment protein (PC) can considerably increase overall hydrothermal liquefaction efficiency [26]. In earlier investigations, authors suggested recycling and transforming PC-extracted *Arthrospira* sp. residue into porous, N-doped graphitic charcoal catalysts to construct an integrated oxidation system for wastewater treatment that would simultaneously remove contaminants and disinfect a given system [27].

Protein hydrolysates are a complex mixture obtained from various protein sources (such as plants and animals) through various techniques, including chemical hydrolysis, microbial fermentation, and enzymatic hydrolysis [28]. With considerable levels of amino acids, macro- and micro-elements, vitamins, and other organic substances, these protein hydrolysates can promote bacterial and fungal growth [21,29,30]. The production of a range of microbial bioactive compounds using residual hydrolysates as low-cost substrates for culture media offers an affordable and sustainable production process. 

Our study presents a new and innovative method for producing melanin using *S. antibioticus* NRRL B-1701. We utilize HAO-DBRH as a cost-effective source of L-tyrosine, which is optimized using RSM. Importantly, this approach has not been previously reported in the literature.

## 2. Materials and Methods

### 2.1. Preparation of the Dried Biomass Residue and Biomass Residue Composition Analysis by Proximate Analysis

*A. platensis* biomass residue after PC extraction was obtained from Greendiamond Co., Ltd., Chiang Mai, Thailand. The drying of the residue was carried out using two different procedures, which included a lyophilizer (Labconco, Kansas, MO, USA) operated at −60 °C and a hot air oven (Binder, Scientific Promotion Co., Ltd., Bangkok, Thailand) operated at 60 °C [31]. The entire drying process required approximately two days to complete. The biomass powders obtained after drying were termed freeze-dried biomass residue (F-DBR) and hot-air-oven-dried biomass residue (HAO-DBR), respectively.

The chemical composition of the F-DBR and the HAO-DBR, including their moisture, ash, lipid, and protein content, were analyzed in the laboratory of Animal Nutrition of the Department of Animal and Aquatic Sciences, Faculty of Agriculture, Chiang Mai University. The analysis was performed according to the Association of Official Analytical Chemists (AOAC) method [32].

### 2.2. Acid-Hydrolysis Treatment

Both F-DBR and HAO-DBR were subjected to acid hydrolysis, with minor modifications, as described in [33]. Shortly, 0.5 g of dried biomass residues was mixed with 15 mL of 3 M HCl (RCl Labscan Limited, Bangkok, Thailand) (1:30 *w*/*v*). The mixture was heated in a water bath for 24 h at 97 °C (Julabo^®^ water bath TW20) and then cooled to room temperature for 30 min. The liquid hydrolysate was separated by centrifugation (MIKRO 220, Hettich Mikroliterzentrifuge, Bangkok, Thailand) at 15,000 rpm for 30 min; then, it was neutralized with CaCO_3_ [24] and filtered through 0.2 µm cellulose acetate (Minisart® Syringe Filter, GIBTHAI, Bangkok, Thailand). This liquid, comprising two components termed freeze-dried biomass residue hydrolysate (F-DBRH) and hot-air-oven-dried biomass residue hydrolysate (HAO-DBRH), respectively, was then stored at −20 °C for further experiments.

### 2.3. Quantitative Analysis of L-tyrosine by Reversed-Phase High-Performance Liquid Chromatography (RP-HPLC)

#### 2.3.1. Pre-Column Derivatization

The L-tyrosine standard (Himedia^TM^, Mumbai, India), F-DBRH, and HAO-DBRH were prepared for HPLC analysis based on the procedures outlined in Kwanyuen and Burton [33] and Santiago-Díaz et al. [34] with slight modifications. To summarize, 1 mL of supernatant was lyophilized using a lyophilizer (Labconco, Kansas, MO, USA) and, subsequently, dissolved in a 1 mL solution consisting of a mixture of ethanol (EtOH) (RCl Labscan Limited, Bangkok, Thailand), DI water, and triethylamine (TEA) (Thermo Fisher Scientific^TM^, Loughborough, UK) in a 2:1:1 ratio. Then, it was combined with 80 µL solution mixture of EtOH, DI water, TEA, and phenylisothiocyanate (PITC) (Sigma-Aldrich^TM^, St. Louis, MO, USA) in a 7:1:1:1 ratio. After allowing the reaction between PITC and the hydrolysate, thereby producing phenylthiocarbamyl (PTC) amino acids, to proceed for 20 min at room temperature, the resulting mixture was filtered through a 0.45 µm nylon filter (Labfil, ALWSCI^®^, Zhejiang, China).

#### 2.3.2. RP-HPLC Analysis

The quantification of L-tyrosine in samples was carried out using the reverse-phase high-performance liquid chromatography (RP-HPLC) method described by Raisa et al. [20] with minor modifications. The analysis was performed using an HPLC system (1200 series, Agilent Technologies, Inc., Santa Clara, CA, USA) equipped with an ultraviolet (UV) detector. The Intersil^®^ ODS-3 column (4.6 × 150 mm, 5 µm) (GL Sciences, Shinjuku-ku, Japan) was used to separate the L-tyrosine. The mobile phase consisted of two solutions: Solution A, which contained 1.4 mM of sodium acetate (RCI Labscan, Bangkok, Thailand), 0.1% trimethylamine, and 6% acetonitrile (RCI Labscan, Bangkok, Thailand) with a pH of 6.1, and Solution B, which was 60% acetonitrile. Both solutions were filtered through a 0.45 µm nylon membrane filter (Filtrex Technologies, Bengaluru, India). The column was eluted using Solution B for 70 min with a linear gradient ranging from 0–100% as described by Park and Oh [35]. The peaks were detected at 254 nm and the standard L-tyrosine was added to the samples (standard addition method) to confirm the peak corresponded to L-tyrosine. The column was regenerated and equilibrated with eluent A for 10 min. A new and freshly reconstituted sample was injected, either by manual injector or autosampler/injector, and analyzed every 70 min.

In the subsequent step, samples with high L-tyrosine concentrations were used to optimize the melanin production medium from the residue. Using the L-tyrosine standard (Himedia^TM^, Mumbai, India), a standard curve was established in the range of 6.25–50 µg, and the L-tyrosine content in each sample was calculated using the following equation:

(1) Y=1305x−745.05

where Y represents the peak area, X represents the concentration of L-tyrosine (µg), and the R-squared value for this formula is 0.9973 (Figure S2).

### 2.4. Optimization of the Melanin Production Medium by Response Surface Methodology (RSM) through Central Composite Design (CCD)

#### 2.4.1. Preparation of the Inoculum and Media

*Streptomyces antibioticus* NRRL B-1701 was preserved in a 20% glycerol solution at −20 °C in the collection of the Microbial Resource and Technology Research Laboratory (MRTRL), Faculty of Science, Chiang Mai University. From this stock culture, *S. antibioticus* NRRL B-1701 was sub-cultured on ISP2 (yeast extract–malt extract) agar plates (Himedia^TM^, Mumbai, India) at 30 °C for 7 days. Subsequently, a single colony was streaked on ISP6 (peptone yeast extract iron) agar plates (Himedia^TM^, Mumbai, India), which were then used as the inoculum and incubated at 30 °C for another 7 days. Five 6 mm mycelial disks from the ISP6 agar plates containing *S. antibioticus* NRRL B-1701 were inoculated into 50 mL of the optimal culture medium within a 125 mL Erlenmeyer flask and incubated at 30 °C with 150 rpm orbital shaking applied (SHEL LAB, Sheldon Manufacturing Inc., Cornelius, OR, USA) [36]. A total of 5% (2.5 mL) of the inoculum was extracted using 5 of the 6 mm disks and then placed in the 50 mL basal medium. The basal medium, with a pH of 6.0, was composed of 5 g/L NaCl, 0.1 g/L CaCl_2_, and four other medium components, namely, yeast extract, soluble starch, HAO-DBRH, and CuSO_4_, which were varied based on the experimental design [37]. All media were incubated for 7 days at 30 °C and centrifuged at 150 rpm.

#### 2.4.2. CCD and RSM

The optimal medium for melanin production by the *S. antibioticus* NRRL B-1701 strain was determined through the usage of RSM. The Design-expert^®^ software (Version 6.0.2; Stat-Ease Inc., Minneapolis, USA.) was employed for both the experimental design and data regression analysis. To determine the individual and combined effects of each operating parameter on melanin yield (g/mL), four independent variables were investigated, including yeast extract (1.40–6.00 g/L), soluble starch (1.00–5.00 g/L), HAO-DBRH (0–37.34 g/L), and CuSO_4_ (0.0072–0.02 g/L) at three different levels (−1, 0, +1), as depicted in Table 1. The levels of each variable were determined (with minor adjustments) based on prior research by Guo et al. [37] and our own preliminary investigations (unpublished data). Table S2 provides all the experimental conditions with coded variables. The primary effects and interactions of each independent variable on the response were evaluated through a second-order polynomial quadratic equation

(2) $$Y=\beta_{0}+\sum \beta_{i}x_{i}+\sum \beta_{ij}x_{i}x_{j}+\sum \beta_{jj\;}x^{2}{}_{j}$$

where Y represents the predicted response, x_i_ and x_j_ represent the variables or parameters, β_0_ represents the offset term, β_i_ represents the linear effect, β_ij_ represents the interaction effect, and β_jj_ represents the squared effect.

The optimal culture broth was analyzed in terms of its melanin content. A total of 1 mL of the culture was collected every 24 h and centrifuged at 10,000 rpm for 15 min to remove the cells and other debris from the medium. The melanin content in the supernatant was quantified spectrophotometrically using a Spectra MR instrument (DYNEX TECHNOLOGIES, Chanilly, VA, USA) at 215 nm [38], with the aid of a standard melanin calibration curve obtained from Sigma-Aldrich^TM^ (St. Louis, MO, USA). The highest melanin concentration from each run was used for further analysis.

Through numerical optimization, several software-proposed solutions were evaluated, and three of the most desirable alternatives were selected for comparison with the suboptimized medium from a previous study [37]. An experimental comparison was performed under these conditions in a shaking incubator, using 250 mL flasks containing 100 mL of sterile medium at pH 6.0 and 30 °C and centrifugation at 150 rpm. The culture broth was monitored every 4 h for 2 days, and the maximum value of absorbance was used to estimate the melanin content.

### 2.5. Cost Analysis

A cost analysis approach was used to determine the cost of the culture medium. The costs of each ingredient required per liter of the medium were obtained from the supplier and calculated. All calculations were performed using Microsoft Excel.

### 2.6. Statistical Analysis

All experimental procedures were replicated three times, and the resulting data are presented as the average value with the corresponding standard deviation. One-way analysis of variance (ANOVA) and Duncan’s multiple range test were employed to determine the significance of the differences in the treatment means. The least significant difference approach, with a significance level of *p* ≤ 0.05, was used to differentiate the means using the Statistical Package for the Social Sciences (SPSS) software version 17.0.

## 3. Results

### 3.1. Preparation of the Dried Biomass Residue and Analysis of Biomass Residue Composition by Proximate Analysis

**Figure 1:**
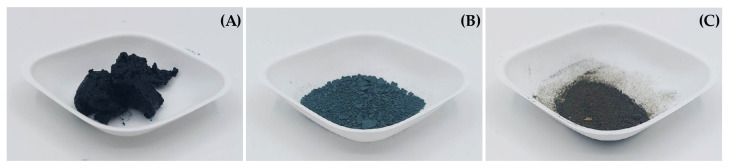
The appearance of *A. platensis* biomass residues: (**A**) the biomass residue after PC extraction; (**B**) F-DBR; and (**C**) HAO-DBR.

The *A. platensis* biomass residue, post-PC extraction, is depicted in Figure 1A, displaying a dark green hue and a moist, sticky mass. The blue-green hue of the dried F-DBR is shown in Figure 1B, while the brown hue of the dried HAO-DBR is shown in Figure 1C, wherein both residues are in the form of dry powder.

Proximate analyses of F-DBR and HAO-DBR are presented in Table 2. Both samples have a high proportion of protein followed by carbohydrates. The levels of moisture, ash, and lipids were similar in both samples.

### 3.2. L-tyrosine Quantitative Analysis by Reversed-Phase High-Performance Liquid Chromatography (RP-HPLC)

**Figure 2:**
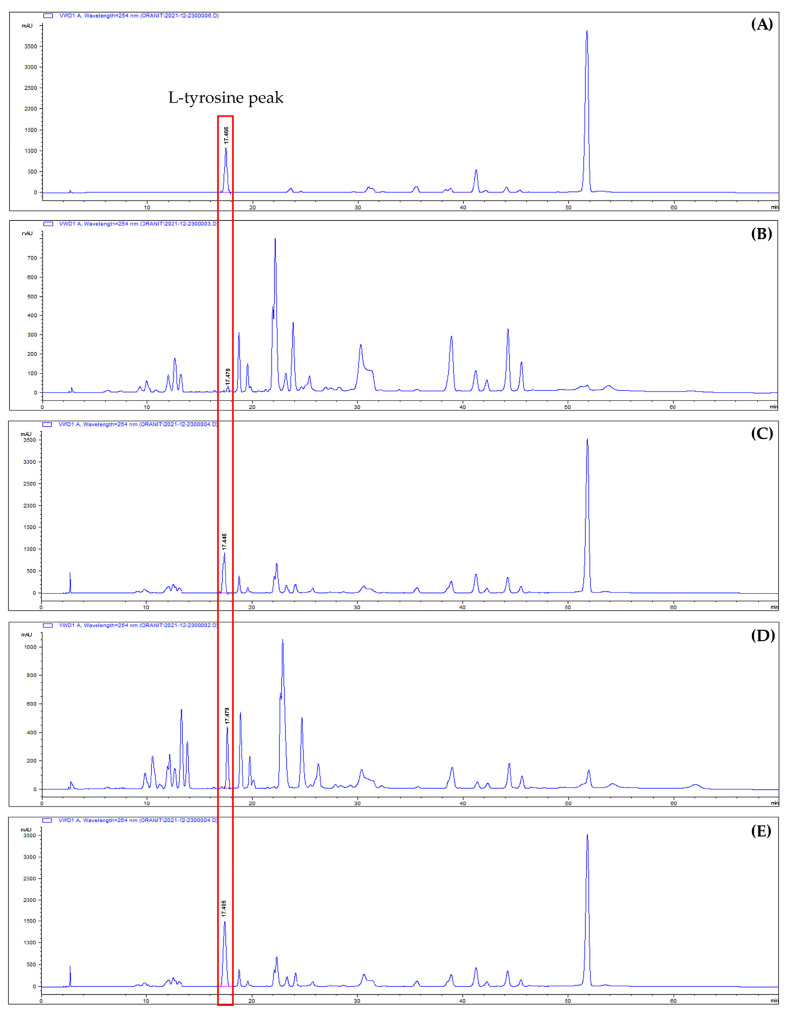
The HPLC chromatogram displays the peak area (mAU) of L-tyrosine in various samples at a retention time of approximately 17.455 ± 0.027 min: (**A**) The standard L−tyrosine (15,635.10 mAU); (**B**) F−DBRH (388.5 mAU); (**C**) F-DBRH mixed with L−tyrosine (16,023.6); (**D**) HAO-DBRH (5091.3 mAU); and (**E**) HAO-DBRH mixed with L−tyrosine (20,726.4 mAU).

The content of L−tyrosine in the F−DBR and HAO-DBR samples was determined by high-performance liquid chromatography (HPLC), for which there was a retention time of 17.455 ± 0.027 min for L−tyrosine, as demonstrated in Figure 2. The concentration of the standard varied from 6.25–50 µg. The chromatograms of the standard L−tyrosine and the graph standard are presented in Figures S1 and S2, respectively. Limit-of-detection (LOD) and limit-of-quantification (LOQ) values are provided in Table S1. The results indicated that the F−DBRH and HAO-DBRH samples contained 0.052 and 0.268 g of L−tyrosine per 100 g of dried biomass, respectively. Based on these results, HAO-DBRH was selected for use in the RSM experimental design due to its higher L-tyrosine content.

### 3.3. Optimization of the Melanin Production Medium by Response Surface Methodology (RSM) through Central Composite Design (CCD)

The optimization of the medium components, including the yeast extract, soluble starch, HAO-DBRH, and CuSO_4_, was carried out to attain the maximum melanin yield. The results of the experiments revealed that melanin production ranged from 0.013 to 0.161 g/L, as presented in Table 3. The CCD experiment demonstrated that the variations in response were due to the diverse concentrations of the components in each experimental group and that the repetitions at the center point resulted in a limited variation range for melanin production.

The response surface model, estimated in the form of a quadratic polynomial regression model, was determined based on the experimental results. The equation is presented in Equation (3), given below:

(3) $$\begin{array}{ll} Y= & -0.026762+0.078051A-0.044377\\ & +5.9659\times 10^{-3}C\;-\;2.59904D\;-\;7.4914\times 10^{-3}A^{2}\\ & +6.7176\times 10^{-3}B^{2}\;-\;1.13692\times 10^{-4}C^{2}\\ & +21.24928D^{2}\;-\;7.60870\times 10^{-4}AB\;-\;4.27913\times 10^{-4}AC\\ & +0.27174AD+3.68238\times 10^{-5}\;BC\\ & +0.52734BD\;-\;0.017784CD \end{array}$$

where *Y* is the predicted melanin production (g/L) and A, B, C, and D are the amounts of yeast extract, soluble starch, HAO-DBRH, and CuSO_4_, respectively.

**Figure 3:**
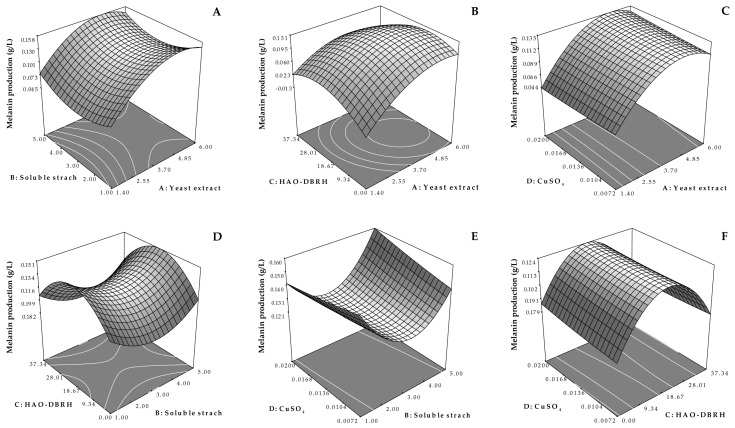
Three-dimensional response surface graphs showing the effect of yeast extract and HAO-DBRH on melanin production: (**A**) yeast extract vs. soluble starch; (**B**) yeast extract vs. HAO-DBRH; (**C**) yeast extract vs. CuSO_4_; (**D**) soluble starch vs. HAO-DBRH; (**E**) soluble starch vs. CuSO_4_; and (**F**) HAO−DBRH vs. CuSO_4_.

The statistical significance of the polynomial model was evaluated through a *t*-test, whose results are presented in Table 4. The analysis of variance (ANOVA) indicated that the polynomial model was highly significant (*p* < 0.01) and the lack of fit was not significant (*p* > 0.05). The coefficient of determination (R^2^) was found to be 0.9472, indicating that 94.72% of the variation in melanin yield could be attributed to the medium components. These results demonstrate that the model was of sufficient quality and accurately represented the relationships between the medium components. Figure 3 presents three-dimensional response surface graphs of the predictive models, of which one was significant while the other was not.

**Figure 4:**
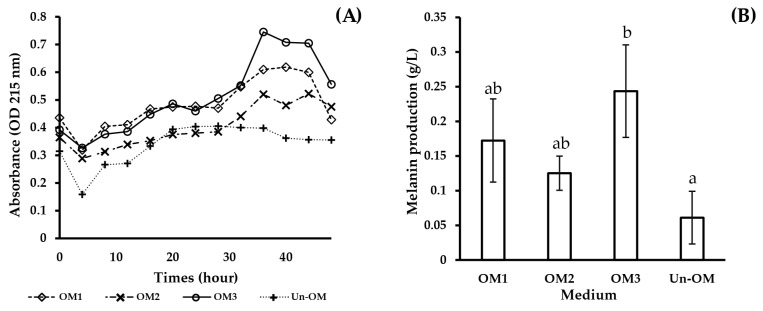
Production of melanin by *S. antiibioticus* NRRL B-1701 in a culture medium: (**A**) Optical density at 215 nm; (**B**) melanin production (g/L) at 36 h. Different lowercase letters indicate significant differences among the four culture mediums in melanin production (*p* < 0.05)

Table 5 displays the three most favorable conditions for melanin production, which were identified using numerical optimization based on the CCD experiment and by comparing the results with those of a reference medium from a previous study [37]. The highest absorbance value was observed in the culture at the 36 h mark, as shown in Figure 4A, and the melanin concentration was measured (shown in Figure 4B).

### 3.4. Cost Analysis

The cost calculation outcomes for each culture medium are presented in Table 6

## 4. Discussion

The PC-extracted residue of *A. platensis* in the study has a dark-green and blue hue due to the remaining PC, as seen in Figure 1A. Drying was used to remove water from solids and thus reduce microbial growth and chemical degradation [39]. The drying methods used in this experiment were lyophilization (freeze drying) and drying via a hot air oven. Lyophilization dehydrates frozen samples by exposing them to low pressure and a temperature of −50 °C, causing ice crystals to sublimate. This method is commonly used for heat-sensitive materials [40], such as PC, a pigment protein with a stability temperature of less than 45 °C [31,41]. A significant amount of PC’s blue color remained after drying using this method, as shown in Figure 1B. The blue color observed in the samples is attributed to the presence of PC, which is a protein pigment found in cyanobacteria. PC is known to be sensitive to high temperatures [42], which can lead to its denaturation. The application of the hot-air-oven-drying method, which involves a temperature of 60 °C, resulted in a loss of the blue color associated with PC, as illustrated in Figure 1C.

The proximate analysis of F-DBR and HAO-DBR was carried out to determine the weight percentage and moisture content of various components such as ash, lipids, proteins, and carbohydrates (Table 2). Both samples showed similar results in terms of moisture content, ash, lipids, and carbohydrates, which were averages of 5.88, 9.56, 5.17, and 12.5, respectively. These findings were consistent with previous reports in the literature [43], which reported moisture content and ash, lipid, and carbohydrate concentrations in the range of 4–7, 6–12, 4–7, and 15–25, respectively.

Protein was the main constituent in both samples, accounting for 69.1% and 68.7% of the total dried biomass in F-DBR and HAO-DBR, respectively. This observation was higher than a previous report that found a protein fraction of 65.6 ± 0.12% in *A. platensis* [44]. The protein content found in this study is also in line with other studies that reported values in the range of 60–70% [45,46,47]. Notably, protein content can vary depending on the algal growth conditions [44]. Despite the removal of PC from the algal biomass, it was discovered that this residue is an excellent source of protein. However, the current work shows a comparable protein concentration of *Arthrospira* sp. that was used for acid hydrolysis.

This study investigated the effect of drying methods on the residue of *A. platensis* after PC extraction and acid hydrolysis. HPLC-UV was used for the quantitative analysis of L-tyrosine. Various techniques are available for amino acid quantification, such as ion-exchange chromatography, gas chromatography (GC), capillary electrophoresis (CE), mass spectrometry (MS), and high-performance liquid chromatography (HPLC). The selection of a method depends on specific analytical requirements, as each technique has its own advantages and disadvantages. HPLC is the most commonly used method for amino acid quantification due to its high sensitivity, specificity, and ability to separate amino acids in complex matrices. For example, Eid et al. [48] used HPLC-UV for the simultaneous quantification of multiple underivatized amino acids in dietary supplements, including isoleucine, leucine, lysine, threonine, histidine, valine, methionine, phenylalanine, tryptophan, and tyrosine. Thus, HPLC is a suitable method for L-tyrosine quantification.

The minimum r^2^ value requirement varies based on the field and purpose of research. The analytical method validation (AMV) protocols we have employed and read about require a minimum correlation coefficient of 0.990 for HPLC-UV [49] and 0.950 for a spectrophotometer [50]. Based on our results, r^2^ = 0.997 is quite acceptable. The findings from the HPLC analysis showed that the L-tyrosine content in the hot-air-oven-dried biomass residue hydrolysate (HAO-DBRH) was higher compared to the freeze-dried biomass residue hydrolysate (F-DBRH). The higher L-tyrosine content in the HAO-DBRH was attributed to the higher temperature used in the hot-air-oven-drying process, which can result in the denaturation of proteins and the release of individual amino acids [51]. Additionally, the denaturation of proteins can cause differences in amino acid profiles, as some amino acids may be more susceptible to hydrolysis than others. Hydrolysis occurs when water is added to a covalent peptide bond, leading to the release of individual amino acids [52]. The complete hydrolysis of proteins to amino acids can occur under high temperatures and acidic conditions [53]. In a neutral pH environment, hydrolysis occurs more slowly and may lead to the formation of peptides and some free amino acids depending on the intensity of the heat treatment. Therefore, the amino acids released from denatured proteins may be either free amino acids or small peptides. It is important to note that the denaturation of proteins can occur without hydrolysis, as the disruption of a protein’s three-dimensional structure does not necessarily break its covalent bonds [52].

Our results are consistent with those obtained in previous research that showed higher amino acid concentrations in dried algae treated at high temperatures, including serine, glycine, histidine, alanine, proline, and L-tyrosine [54]. The amount of L-tyrosine in HAO-DBRH was slightly lower than that found in the initial report by AlFadhly, N.K. et al. [42], who reported 0.3 g of L-tyrosine/100 g dried biomass. However, the L-tyrosine obtained in this study was greater than some previous findings claiming that *Arthrospira* sp. contained between 0.186 and 0.160 g of L-tyrosine per 100 g of dried biomass [52,53]. L-tyrosine content can differ between different algal species and depend on various environmental factors, including temperature, light, nutrients, extraction techniques, and processing. The findings of this study suggest that the hot-air-oven-drying method can be employed to increase the L-tyrosine content in *Arthrospira* sp. residues. It was concluded that HAO-DBRH would be the preferred sample for further experiments due to its higher L-tyrosine content.

To optimize the production of melanin using L-tyrosine from the hydrolysate of *A. platensis* residues, this study employed the use of RSM as a statistical experimental design technique. RSM is an effective tool for simultaneously optimizing medium components, such as yeast extract, soluble starch, HAO-DBRH, and CuSO_4_, and evaluating their interactions. The application of RSM can provide mathematical models that aid in understanding the interplay between different levels of parameters and how to achieve the optimal level of each parameter to achieve a specific goal [54]. This approach to optimization offers several benefits, such as reducing manufacturing times, saving operation costs, and improving output quality. This study successfully used RSM as a strategy for improving an melanin production medium to obtain the highest yield of melanin from *S. antibioticus* NRRL B-1701.

The results obtained from this study, as presented in Table 4, demonstrate a highly significant (*p* < 0.01) quadratic effect of yeast extract and HAO-DBRH on melanin production, while the other factors were not significant. The effect of yeast extract and HAO-DBRH on melanin yield in the optimum medium when the concentrations of soluble starch and CuSO_4_ were maintained at 3.0 and 0.0136 g/L, respectively, are illustrated in Figure 3B. The results show that when the concentration of HAO-DBRH was constant, melanin production was increased through the incorporation of a 4.85 g/L concentration of yeast extract but remained unchanged thereafter. This finding is supported by previous studies that suggested the use of yeast extract as a nitrogen source in a medium to improve pigment synthesis in *Streptomyces chibaensis* [55]. Additionally, the use of yeast extract as a source of nitrogen for marine *Streptomyces* strains that produce melanin has been explained in another study [56].

Moreover, when the concentration of yeast extract remained constant, an increase in HAO-DBRH concentration increased melanin yield, but gradually decreased thereafter. The maximum value of melanin yield was attained when the concentration of HAO-DBRH was around 18.67 g/L, indicating that excess HAO-DBRH might reduce melanin yield. This result could be attributed to the fact that some amino acids (such as L-tyrosine) released during the hydrolysate residue process [21] can encourage the formation of melanin and serve as a substrate in the melanin synthesis pathway [57,58]. This finding is in agreement with that found by Zou and Hou [36], who demonstrated that L-tyrosine can enhance melanin production but has the opposite effect when applied in excess due to a decrease in the solubility of L-tyrosine at higher concentrations, which leads to the accumulation of the substrate in the reactor by precipitation. This accumulation can cause disruptions in the normal functioning of cells as the degradation of L-tyrosine releases ammonia [59]. Hydrolysate is a complex mixture of oligopeptides, peptides, and free amino acids [27]. However, the presence of inhibitory compounds in hydrolysates that may negatively affect microbial melanin production should be taken into consideration. Therefore, further studies are necessary to identify these compounds and evaluate their impacts on melanin production in order to obtain a more comprehensive understanding of the effects of hydrolysates in this process.

Therefore, the results of this study provide a better understanding of the effect of yeast extract and HAO-DBRH on melanin production, which can inform the optimization of medium components and contribute to the development of more efficient and cost-effective melanin production processes.

As a result of RSM optimization, the maximum level of melanin production was the highest in OM3 medium, which contained yeast extract, soluble starch, CuSO_4_, and HAO-DBRH concentrations of 5.16, 4.92, 0.0191, and 17.11 g/L, respectively. The maximum melanin production level predicted by the quadratic model was 0.164 g/L, which was verified in a validation experiment. The experimentally observed maximum melanin production level was 0.244 g/L, which could be attributed to the continuous monitoring of the absorbances at four-hour intervals, whereby the highest absorbance value was detected at 36 h.

Time is a crucial aspect in the fermentation process as a pigment can be degraded by light [15]; therefore, melanin functions as a photoprotective pigment or can be excreted by microbes from the fermentation medium [17]. This result differs from the findings of previous investigations [15,38,60], which demonstrated that the highest melanin yield was detected after seven days. However, when comparing the degree of melanin production at 36 h, the optimized medium produced more melanin than the unoptimized medium according to Guo et al. [37], with OM3 being the most effective, yielding around four times more melanin than the control.

The cost analysis of the medium used in the experiment was carried out based on the price of the ingredients and is presented in Table 6. The cost of the optimized medium that produced the highest amount of melanin was USD 0.52 per gram of melanin. This is significantly less expensive compared to the previously used medium [37], which cost USD 10.20 per gram of melanin. The use of HAO-DBRH as an ingredient in the medium was found to be a cost-effective alternative to chemical L-tyrosine and resulted in an increase in melanin production by *S. antibioticus* NRRL B-107 and a reduction in the overall cost of the medium.

Studies have highlighted the significance of using non-animal protein sources in the pharmaceutical industry and the usage of protein hydrolysates from *A. platensis* as a safe and effective alternative to peptone in microbial cultures. This aligns with the United Nations’ sustainable development goals (SDGs), which concern the use of natural and renewable resources to develop a bioeconomy [61], emphasizing the importance of natural resources in achieving a sustainable future. Thus, the use of low-cost and sustainable ingredients such as HAO-DBRH in a medium is an essential step toward a more sustainable and eco-friendly approach to melanin production.

Our current research is focused on optimizing the batch system’s performance. Nevertheless, we recognize the potential benefits of using a fed-batch system, which can reduce the accumulation of byproducts and improve overall efficiency. Therefore, we plan to investigate the feasibility of this optimization approach in a fed-batch system in future research. Our goal is to contribute to sustainable development by promoting efficient and sustainable bioprocessing methods through our research.

## 5. Conclusions

The study evaluated the potential of using an *A. platensis* residue hydrolysate as a tyrosine source to potentiate melanin production by *S. antibioticus* NRRL B-107. The proximate analysis of the dried biomasses after PC extraction showed that a high percentage of protein remained. Of the two hydrolysates tested, HAO-DBRH had a higher concentration of L-tyrosine, thus rendering it the preferred source for optimization. Using the RSM methodology, optimal culture conditions were determined, leading to a melanin yield of 0.24 g/L in 36 h, which constitutes a four-fold increase compared to the non-optimized medium. This study provides evidence that the hydrolysate of PC-extracted *A. platensis* can be used as a low-cost source of L-tyrosine for melanin production by actinobacteria, which has demonstrated its potential as a sustainable and cost-effective alternative for use in the pharmaceutical industry.

## Figures and Tables

**Table 1:** Factors and levels in the experimental design.

Factor	Level		
	−1	0	1
Yeast extract (g/L)	1.40	3.7	6.00
Soluble starch (g/L)	1.00	3.0	5.00
HAO-DBRH (g/L)	0	18.67	37.34
CuSO_4_ (g/L)	0.0072	0.0136	0.02

**Table 2:** Proximate composition of *A. platensis* dried biomass residue after PC extraction.

Composition	Samples	
	F-DBR	HAO-DBR
Moisture (%)	5.92	5.83
Ash (%)	7.38	7.73
Lipids (%)	5.20	5.13
Proteins (%)	69.1	68.7
Carbohydrates (%)	12.4	12.61
Total composition (%)	100	100

**Table 3:** CCD matrix and response values.

Run	Actual Variables				Actual Responses
	Yeast Extract (g/L)	Soluble Starch (g/L)	HAO-DBRH (g/L)	CuSO_4_ (g/L)	Melanin Production (g/L)
1	1.4	5.0	0.0	0.02	0.013
2	1.4	5.0	0.0	0.0072	0.016
3	3.7	3.0	18.67	0.0072	0.123
4	3.7	1.0	18.67	0.0136	0.161
5	6.0	5.0	37.34	0.02	0.097
6	6.0	5.0	0.0	0.02	0.134
7	6.0	1.0	37.34	0.0072	0.106
8	3.7	3.0	0.0	0.0136	0.098
9	6.0	5.0	37.34	0.0072	0.074
10	3.7	5.0	18.67	0.0136	0.150
11	3.7	3.0	37.34	0.0136	0.080
12	6.0	1.0	37.34	0.02	0.078
13	3.7	3.0	18.67	0.0136	0.095
14	1.4	5.0	37.34	0.0072	0.053
15	6.0	1.0	0.0	0.0072	0.108
16	1.4	1.0	37.34	0.02	0.027
17	3.7	3.0	18.67	0.0136	0.118
18	6.0	3.0	18.67	0.0136	0.130
19	6.0	5.0	0.0	0.0072	0.116
20	1.4	5.0	37.34	0.02	0.076
21	1.4	1.0	0.0	0.0072	0.021
22	3.7	3.0	18.67	0.02	0.136
23	1.4	3.0	18.67	0.0136	0.048
24	1.4	1.0	0.0	0.02	0.004
25	3.7	3.0	18.67	0.0136	0.112
26	6.0	1.0	0.0	0.02	0.134
27	1.4	1.0	37.34	0.0072	0.055

**Table 4:** ANOVA for response surface quadratic model of response variance.

Source	SS	Df	MS	F-Value	*p* > F
Model	0.05	14	3.545 × 10^−3^	15.38	<0.0001
A: Yeast extract	0.024	1	0.024	106.27	<0.0001
B: Soluble starch	6.806 × 10^−5^	1	6.806 × 10^−5^	0.30	0.5968
C: HAO-DBRH	2.222 × 10^−7^	1	2.222 × 10^−7^	9.642 × 10^−4^	0.9757
D: CuSO_4_	4.050 × 10^−5^	1	4.050 × 10^−5^	0.18	0.6825
A^2^	4.038 × 10^−3^	1	4.038 × 10^−3^	17.52	0.0013
B^2^	1.857 × 10^−3^	1	1.857 × 10^−3^	8.06	0.0149
C^2^	4.038 × 10^−3^	1	4.038 × 10^−3^	17.52	0.0013
D^2^	1.948 × 10^−6^	1	1.948 × 10^−6^	8.452 × 10^−3^	0.9283
AB	1.960 × 10^−4^	1	1.960 × 10^−4^	0.85	0.3746
AC	5.402 × 10^−3^	1	5.402 × 10^−3^	23.44	0.004
AD	2.560 × 10^−4^	1	2.560 × 10^−4^	1.11	0.3127
BC	3.025 × 10^−5^	1	3.025 × 10^−5^	0.13	0.7234
BD	7.290 × 10^−4^	1	7.290 × 10^−4^	3.16	0.1007
CD	7.225 × 10^−5^	1	7.225 × 10^−5^	0.31	0.5859
Residual	2.766 × 10^−3^	12	2.766 × 10^−3^		
Lack of Fit	2.481 × 10^−3^	10	2.481 × 10^−3^	1.74	0.4190
Pure Error	2.847 × 10^−4^	2	1.423 × 10^−3^		
Cor Total	0.052	26			
R^2^	0.9472				
C.V.	17.35				

Significant within a 95% confidence interval; SS—sum of squares; Df—degree of freedom; MS—mean square; R^2^—regression coefficient; C.V.—coefficient of value.

**Table 5:** Medium compositions.

Conditions	Yeast Extract (g/L)	Soluble Starch (g/L)	CuSO_4_	NaCl (g/L)	CaCl_2_ (g/L)	HAO-DBRH	Predicted Value	Actual Value
			(g/L)			(g/L)	(g/L)	(g/L)
OM1	4.65	4.98	0.0179	5	0.1	17.89	0.164	0.172
OM2	4.14	4.98	0.0198	5	0.1	17.30	0.165	0.125
OM3	5.16	4.92	0.0191	5	0.1	17.11	0.164	0.244
Un-OM	37	3.3	0.0136	5	0.1	-	-	0.061

OM1-3—three of the most desirable alternatives obtained from numerical optimization; Un-OM—a non-optimization medium.

**Table 6:** Comparison of medium cost.

	OM1	OM2	OM3	Un-OM
Yeast extract	0.74	0.66	0.82	5.86
Soluble starch	0.25	0.25	0.25	0.17
CuSO_4_	0.0001	0.00012	0.00011	0.00008
NaCl	0.195	0.195	0.195	0.195
CaCl_2_	0.004	0.004	0.004	0.004
HAO-DBRH	0	0	0	0
HCl	8.82	8.82	8.82	0
Medium cost (USD/L)	1.19	1.11	1.25	6.22
Experimentally melanin production (g/L)	1.72	1.25	2.44	0.61
Medium cost (USD/g melanin)	0.69	0.89	0.52	10.20

OM1-3—three of the most desirable alternatives obtained from numerical optimization; Un-OM—a non-optimization medium. This price was calculated on February 1, 2023, according to the exact price listed by the chemical manufacturer.

## Data Availability

All data underlying the results are included as part of the published article and its Supplementary Materials.

## Supplementary Materials

The following supporting information can be downloaded at: https://www.mdpi.com/article/10.3390/biotech12010024/s1, Figure S1: Chromatogram shows peak area (mAU) of standard L-tyrosine (retention time around 17.437 ± 0.029) detected by high-performance liquid chromatography (HPLC) after being derivatized with phenylisothiocyanate (PITC) solution.; Figure S2: Calibration curve of L-tyrosine; Table S1: Limit of detection (LOD) and limit of quantification (LOQ); Table S2: CCD matrix in terms of coded variables.
