# Supplementary material for: Optimization of Melanin Production by Streptomyces antibioticus NRRL B-1701 Using Arthrospira (Spirulina) platensis Residues Hydrolysates as Low-Cost L-tyrosine Supplement
Source: BioTech (Basel). 2023 Mar 20;12(1):24. doi: 10.3390/biotech12010024 (PMC10046677; doi:10.3390/biotech12010024)
Supplement: Supplementary file 1 [file biotech-12-00024-s001.zip › biotech-2263379-supplementary.docx]

*Supplementary Material*

**Optimization of Melanin Production by *Streptomyces antibioticus* NRRL B-1701** **Using *Arthrospira (Spirulina) platensis* Residues Hydrolysates as Low-cost L-tyrosine Supplement**

Oranit Kraseasintra, Sritip Sensupa, Kanjana Mahanil, Sada Yoosathaporn, Jeeraporn Pekkoh, Sirasit Srinuanpan, Wasu Pathom-aree, Chayakorn Pumas


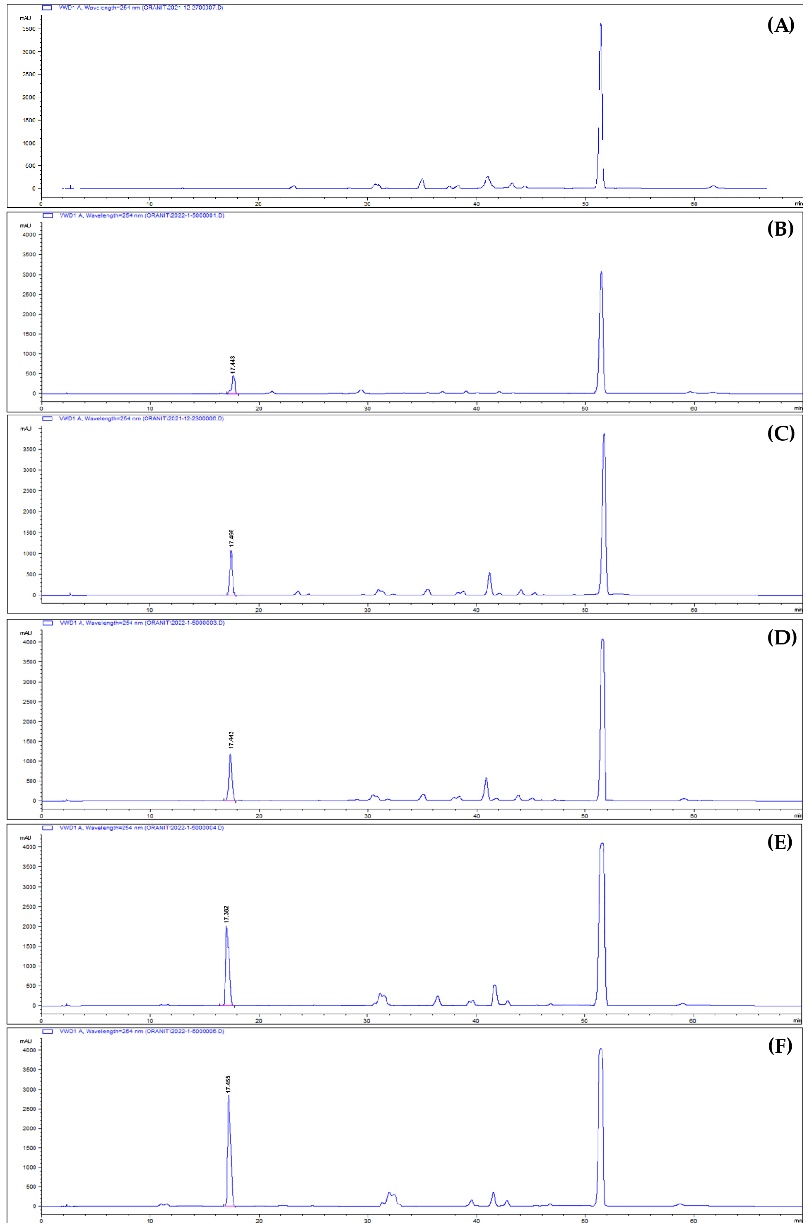


**Figure S1.** Chromatogram showed peak area (mAU) of standard L-tyrosine (retention time around 17.437±0.029) detected by high-performance liquid chromatography (HPLC) after being derivatized with phenylisothiocyanate (PITC) solution.; (A) blank (B) standard L-tyrosine 6.25 µg (7503.7 mAU); (C) standard L-tyrosine 12.5 µg (15635.10 mAU); (D) standard L-tyrosine 25 µg (30664.0 mAU); (E) standard L-tyrosine 37.5 µg (50,098.0 mAU); and (F) standard L-tyrosine 50 µg (63,655.5 mAU).


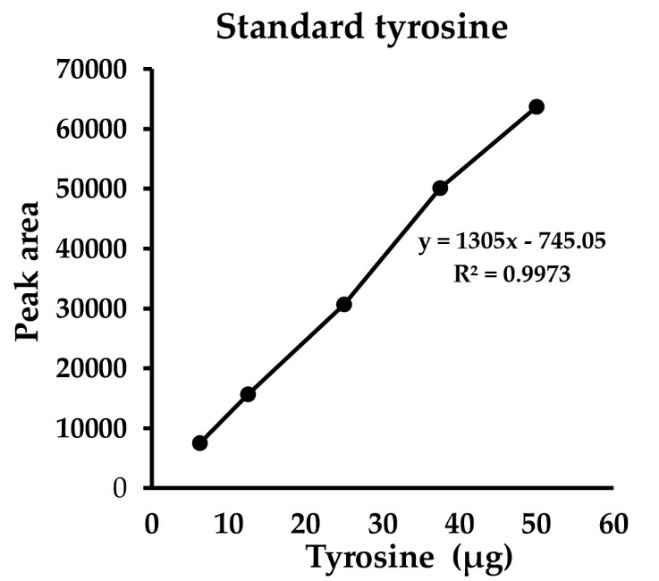


**Figure S2.** Calibration curve of L-tyrosine

**Table S1.** Limit of detection (LOD) and limit of quantification (LOQ)

| **Validation factors** | **Value** |
| --- | --- |
| SE of intercept  SD of intercept  LOD  LOQ  √N  Slop | 1198.8873  2680.7935  0.14  0.41  2.2360  1305 |

**Table S2.** CCD matrix in term of coded variables

| **Run** | **Actual variables** | | | |
| --- | --- | --- | --- | --- |
|  | **Yeast extract (g/L)** | **Soluble starch (g/L)** | **HAO-DBRH (g/L)** | **CuSO_4_ (g/L)** |
| 1  2  3  4  5  6  7  8  9  10  11  12  13  14  15  16  17  18  19  20  21  22  23  24  25  26  27 | -1  -1  0  0  1  1  1  0  1  0  0  1  0  -1  1  -1  0  1  1  -1  -1  0  -1  -1  0  1  -1 | 1  1  0  -1  1  1  -1  0  1  1  0  -1  0  1  -1  -1  0  0  1  1  -1  0  0  -1  0  -1  -1 | -1  -1  0  0  1  -1  1  -1  1  0  1  1  0  1  -1  1  0  0  -1  1  -1  0  0  -1  0  -1  1 | 1  -1  -1  0  1  1  -1  0  -1  0  0  1  0  -1  -1  1  0  0  -1  1  -1  1  0  1  0  1  -1 |
